# The multifaceted impact of stress on immune function

**DOI:** 10.1007/s11033-025-11134-6

**Published:** 2025-10-10

**Authors:** Marta Balcerowska, Paulina Kwaśnik

**Affiliations:** Department of Experimental Hematooncology, Medical University of Lublin, Lublin, Poland

**Keywords:** Acute stress, Cancer, Chronic stress, Immune system, Stressors

## Abstract

Stress is a response to physiological or psychological threats or challenging situations that enable one to cope with difficult circumstances. Various types of stressors promote a coordinated response in the body to maintain homeostasis. Experimental evidence suggests that the duration of stress is a pivotal factor influencing dysregulation in the immune system caused by stress. While it was reported that acute stress prepares the organism to deal with challenges and can benefit the body, chronic stress is detrimental. It can lead to more frequent infections, prolonged inflammation, and even cancer progression. This review aims to identify the consequences of stress on the immune system, distinguishing between acute stress and chronic stress. We conducted a comprehensive literature review to investigate how different types of stressors and the duration of stress affect the immune system. Both acute and chronic stress have an impact on the immune system. In the short term, stress induces the redistribution of immune cells by mobilizing them to active defense, and promotes anti-infective and anti-tumor defense, whereas chronic stress deregulates the immune system by reducing the effectiveness of immune cells, affecting their ability to fight infections and increasing the rate of disease progression.

## Introduction

Stress is an integral part of human life. It encompasses the difficult circumstances individuals face, leading to physiological and psychological responses to these obstacles. Stress affects various systems in the body, and one that is significantly influenced is the immune system. Based on the duration of stress, it can be divided into acute stress and chronic stress [1]. If stress lasts a few minutes, it is defined as acute stress. It can mobilize an organism to create a response that will enable survival, the so-called “fight or flight” response. Another form of stress is chronic stress, which occurs after exposure to stressors over an extended period. Studies have shown that this type of stress has deleterious effects on the immune system [1, 2]. The initial model, which considered the relationship between stress and the immune system, postulated the immunosuppressive effects of stress [2]. Chronic stress impacts circulating inflammatory markers, leading to persistent low-grade inflammation, which modulates immune response [3]. Inflammation is the biological response of the immune system to the effects of invading external pathogens or endogenous biomolecules. The persistent inflammatory response is associated with organ dysfunction and pathology. Increased levels of systemic interleukin-6 (IL-6) and C-reactive protein (CRP) are observed during inflammation and have also been observed in response to stress [3]. Consequently, negative changes in the immune system increase the risks and severity of infectious disease and might even be a key factor for tumor development and progression.

However, over time, the validity of this model has been questioned, considering the adaptive response to life-threatening circumstances triggered by short-term stressors. Experimental evidence suggests that the duration of stressors decisively determines their impact on the functioning of the immune system, contributing to changes in the number and function of B, T, and natural killer (NK) cells after exposure to stress. Based on these findings, a two-phase model was proposed, in which acute stress enhances and chronic stress suppresses the immune response. Acute, time-limited stressors—often consistent with the “fight or flight” paradigm—are believed to induce an adaptive redistribution of immune cells to compartments where they can function most rapidly and effectively [4]. Interestingly, current models have gone a step further, considering the division of the immune system into innate and adaptive components - and within the adaptive system, into cellular and humoral components - which provides a useful framework for interpreting the differential effects of stressors, taking into account the energy expended to mount an effective immune response. Acute stressors enhance innate immune parameters while transiently inhibiting some aspects of adaptive immunity. This shift has been interpreted as an energy-saving strategy, preparing the body for potential injury or infection by prioritizing rapid, innate defense mechanisms [5]. Acute stress has been shown to increase the number and activity of innate immune cells while simultaneously reducing the proliferative responses of adaptive lymphocytes, which require a greater investment of time and energy.

Chronic or long-term stressors, in turn, are associated with global immunosuppression and functional impairment, particularly in the adaptive arm of immunity. This is reflected in reduced lymphocyte proliferation, impaired cytokine production, and a shift toward functional exhaustion of T and B lymphocyte responses. Therefore, while short-term stress can transiently enhance host defenses through innate mechanisms, long-term stress impairs both innate and adaptive immunity, contributing to increased susceptibility to infection and impaired immune surveillance.

However, the findings linking chronic stress with the potential development of diseases, especially cancer, remain an unmet need for clarification. One recent model, which considers patterns of immunological changes as well as patterns of stress-related disease, posits that chronic stress induces simultaneous enhancement and suppression of the immune response through altered cytokine secretion patterns [6]. This explains the discrepancies in the literature regarding the effects of stress on individual immune populations. Prolonged exposure to a stressor induces cortisol release, which leads to a shift in the immune response from Th1 to Th2. Reduced Th1-dependent cellular immune responses may increase susceptibility to infectious and neoplastic diseases, while increased Th2-dependent humoral immune responses can increase susceptibility to autoimmune and allergic diseases [7]. Current models of stress-immune system interactions emphasize that the duration of stressors crucially determines their impact on immune function, and even when the stressor is initially acute and short-lived, the potential adaptive power of immune changes decreases with prolonged exposure. However, the redundancy and overlap between innate and adaptive responses underscore the need for integrative models that consider simultaneous activation and regulation in various immune domains.

Various types of stressors are presented, and they can be broadly categorized into physical and psychological stressors [1]. Physical stressors include pathogens and external circumstances, like loud noise or extreme temperatures. Importantly, also intense physical exercise can act as a stressor, as it triggers neuroendocrine activation and transient physiological changes that resemble a stress response. The short-term physical stress can enhance the immune system by promoting the mobilization and redistribution of lymphocytes [8, 9]. In contrast, psychological stressors are challenging circumstances for the organism, such as interpersonal conflicts or significant life changes, that require a person to adapt and respond appropriately to maintain well-being. These stressors often arise from perceived threats to safety or stability, and their effects extend beyond the mental realm and can induce a prolonged stress response, which, if persistent, leads to physiological changes and health consequences. Long-term psychological stress impairs the immune system’s response, promoting chronic inflammation, which can contribute to lymphocyte exhaustion [1].

Nevertheless, not every stressor induces identical responses across individuals. Biological sex, genetic factors, physiological reactivity, and the presence of comorbidities all contribute to variability in stress responses. In particular, sex-related differences in the hypothalamic–pituitary–adrenal (HPA) axis and sympathetic nervous system reactivity can lead to divergent responses between men and women. For instance, men often display a stronger cortisol increase after acute psychological stress, whereas in women the magnitude and direction of cortisol responses are strongly influenced by sex hormones, age, and menstrual cycle phase [10, 11]. These biological differences may contribute to the higher prevalence of stress-related mood and anxiety disorders observed in women, underlining the importance of considering sex as a moderator of stress response.

Genetic differences also play an important role in creating individual stress responses. Research among inbred mouse strains revealed individual differences in the level of circulating corticosterone (CORT) after exposure to an acute stressor, reflecting genetic influences on HPA axis reactivity. There were significant differences in organ weight and behavioral response among strains [12]. Additionally, investigating *ADRB2* polymorphism, which encodes β2-adrenergic receptor (β2-AR), can provide insight into genetic contributions to stress-induced cardiovascular changes. A study involving young men demonstrated that the *rs1042713* and *rs1042714* variants were associated with increased blood pressure in response to a stressor. It was observed that athletes and non-athletes with the *rs1042713* polymorphism had different levels of systolic blood pressure (SBP), but among the athlete group, heightened blood pressure appeared to be influenced mostly by the *rs1042714* polymorphism. Furthermore, *rs1042713* homozygotes showed a more pronounced rise in SBP [13]. Given that blood pressure regulation results from complex genetic–environmental interactions, these findings provide only limited insight into the precise role of *ADRB2* polymorphism in stress reactivity.

Physiological markers such as heart rate variability (HRV) have also been identified as indicators of individual differences in stress reactivity. Stronger HRV reactivity was correlated with momentary emotional exhaustion, suggesting that individuals with heightened physiological reactivity may be more vulnerable to emotional depletion in daily life than individuals whose HRV was weaker in response to stress [14]. A prospective clinical study indicated that factors related to stress contribute to the development of cardiovascular disease (CVD). Participants with elevated cortisol levels in response to acute mental stress predicted the later development of hypertension, linking stress-induced HPA axis activation to risk of CVD [15]. These studies convincingly demonstrate that people’s cardiovascular and neuroendocrine responses to stressful experiences depend on their appraisal of the situation and subjective experience, which also makes a significant contribution to explaining individual differences in stress response.

Additionally, the presence of comorbidities was associated with exacerbated stress response and risk of anxiety. Individuals with chronic disease report higher levels of perceived stress, anxiety, and depression. This highlights the bidirectional relationship between stress and chronic illnesses, where comorbidities not only amplify stress responses but also increase vulnerability to mental health disorders. These studies highlight that stress responses are highly heterogeneous. Recognizing such individual differences is essential for understanding the pathways through which stress contributes to both physical and mental health outcomes.

This review explores the key role of stress and its implications for immunity, emphasizing the importance of prolonged stress duration in the induction of immune dysfunction. Furthermore, the review provides a comprehensive overview of the types of stressors, the mechanisms generated in response to different stressors, the differences between acute and chronic stress, and their consequences, especially in the context of the immune system. In the era of the continuing need to explain the etiology of cancer, in this review, we emphasize the role of stress in carcinogenesis.

### Hypothalamic–pituitary–adrenal axis

Stress can have beneficial effects on systems in the human body. In response to stressors, it triggers multiple homeostatic mechanisms to protect one’s body from threats. The HPA axis and the sympathetic-adrenal-medullary (SAM) are the main components that induce the stress response. HPA activation results in a release of corticotropin-releasing factor (CRF), which triggers the release of adrenocorticotropic hormone (ACTH) via binding to its receptors. Afterward, ACTH binds to its receptors and stimulates the adrenal release of cortisol, referred to as the “stress hormone”, which plays a crucial role in modulating immune responses. This hormone promotes physiological and behavioral responses to stress by increasing glucose levels as an energy source and modulating the cardiovascular capacity to ensure suitable perfusion of vital organs in response to a potential threat. Moreover, cortisol influences cognition to shape appropriate behavioral responses to stress. Consequently, activation of HPA and SAM will result in changes in multiple systems in the body at one time In the context of the immune system, the HPA axis stimulates its function by secreting glucocorticoids and mineralocorticoids into the bloodstream. These hormones bind to specific receptors expressed on immune cells, influencing their activity. It was shown that macrophages can release IL-6 as a response to activation of adrenergic receptors (ARs), which in turn leads to redistribution of leukocytes [16].

### Effects of acute stress on the immune system

The role of acute stress is to induce a rapid response to various stressors to restore homeostasis. The key regulatory pathway to maintain homeostasis in response to stress is the HPA axis. During the initial phase of the stress response, the increase in cortisol contributes to an increase in the number of circulating B lymphocytes, enhancing the adaptive immune response and the activity of specific immune cells such as NK cells. This, in turn, triggers the production of pro-inflammatory cytokines, including IL-6 and tumor necrosis factor (TNF). The main role of IL-6 is to induce inflammation and also stimulate hepatocytes to release acute-phase response proteins. TNF influences endothelial permeability to ensure optimal transport of immune cells to peripheral tissues where inflammation occurs. Most clinical and translational studies to date have disproportionately focused on IL-6 and TNF as prototypical pro-inflammatory markers. While these cytokines are undoubtedly important, this narrow focus risks overlooking other mediators such as interleukin 1 beta (IL-1β), granulocyte-macrophage colony-stimulating factor (GM-CSF,) and chemokines including CCL2 and CXCL8, which play key roles in immune cell recruitment and tumor–immune interactions. A broader cytokine profiling approach is therefore needed to fully capture the immune landscape associated with chronic stress. Studies have shown that exposure to stress hormones can change the redistribution of immune cells by directing leukocyte subpopulations to specific organs to prepare the body for a needed immune reaction. Stress mediators such as norepinephrine (NE) induce trafficking of specific subpopulations to the target organs, providing a protective immune response by increasing the number of cytotoxic T lymphocytes (CTLs) and NK cells activation [17]. The study by Viswanathan et al. [18] demonstrated that acute stress significantly increases the infiltration of various leukocyte subpopulations (neutrophils, macrophages, NK cells, B and T cells), temporarily raising the number of immune cells in circulation. This ensures proper functioning of immune surveillance.

### Impact of physical exercise on the immune system

Physical exercise is a unique type of stressor that has a beneficial impact on certain aspects of immunity, depending on its intensity and duration. During exercise, eccentric contractions induce mechanical stress that damages activated fibers. To support the heightened activity and meet the oxygen demands of working muscles, the blood flow is increased, the heart rate is elevated, and the level of cortisol rises, resulting in physiological changes similar to those observed during a stress response. Regular and moderate physical activity can prevent the development of chronic diseases and reduce the risk of cancer, mostly by promoting an anti-inflammatory status [7]. The study by Chen et al. [9] demonstrated that mice exposed to regular exercise developed an adaptive immune response, establishing a relatively anti-inflammatory state compared to mice not exposed to regular exercise. They proposed that elevated levels of glucocorticoids after exercise may increase expression of mitogen-activated protein kinase phosphatase 1 (MKP-1), which prevents excessive inflammatory responses by negatively regulating mitogen-activated protein kinase (MAPK). This suggests that physical exercise may increase MKP-1 expression and reduce excessive inflammatory responses by mitigating MAPK activation.

Exercise modulates the immune system by altering the number and distribution of immune cells. Regular physical activity is able to increase the number of lymphocytes in the long term, an effect that may be partly mediated by elevated catecholamine levels [8]. However, several hours after exercise, peripheral blood lymphocyte counts have been observed to decline below pre-exercise levels, creating a short-lived window of immunosuppression. NK cells appear particularly sensitive to this effect, as their levels in peripheral blood decrease following intense exercise [8]. Importantly, post-exercise lymphopenia may not be harmful; rather, it is hypothesized to support immune regulation, as it may result from redistribution of immune cells to peripheral tissues, enhancing immune surveillance [1]. In this context, the observed reduction in circulating lymphocytes reflects a temporary, heightened state of immune surveillance and immune regulation driven by a preferential mobilization of cells to peripheral tissues [7].

### Fever and its immunological implications

**Fig. 1:**
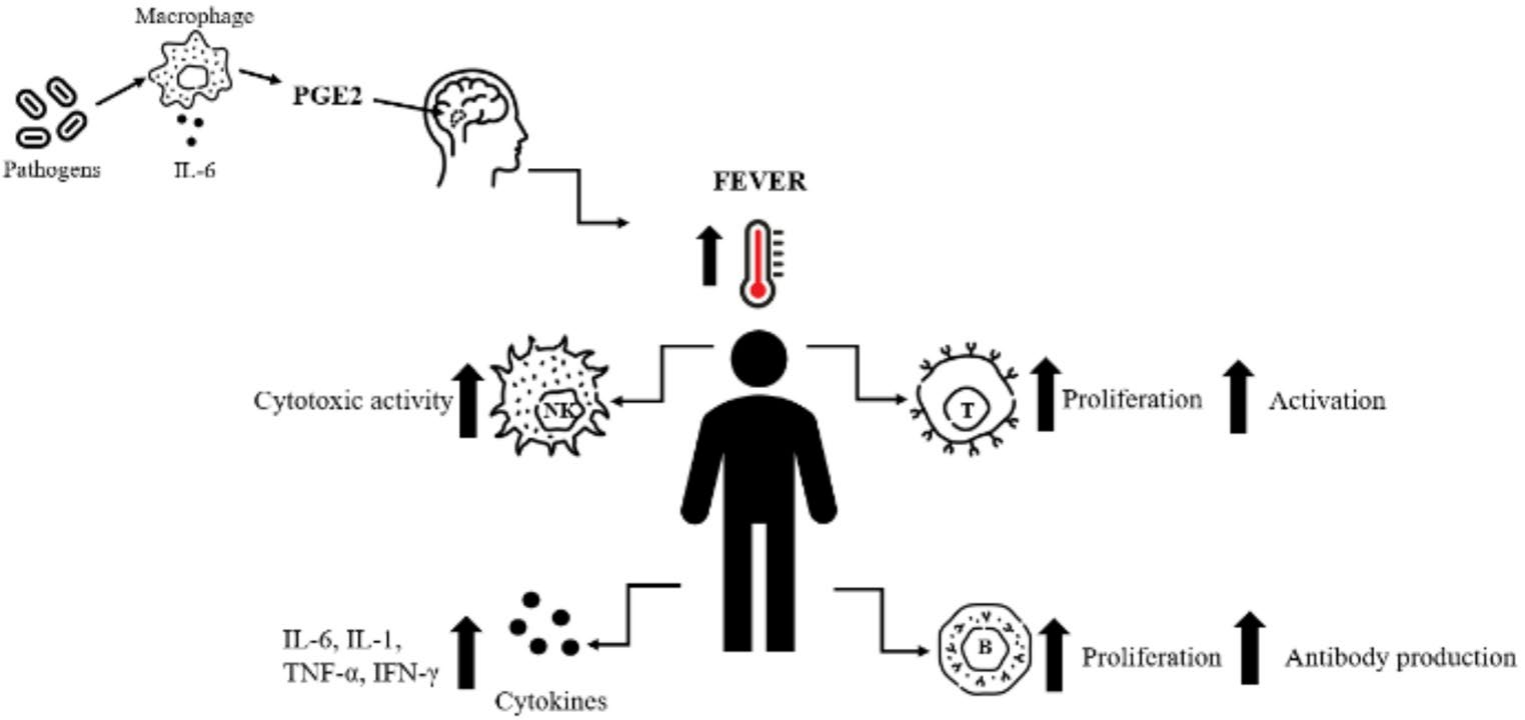
The activation of the fever-induced immune response. The figure shows the pathways of immune response activation due to fever. In the presence of pathogens, activated macrophages release IL-6 and its elevated levels are additionally induced by the production of PGE2 in the hypothalamus. This cascade of actions triggers fever. The increased body temperature enhances immune cells proliferation, activation and also stimulates the release of cytokines: IL-6, IL-1 β, TNF, interferon gamma (INF-γ). T cells undergo proliferation and activation, B cells increase antibody production, and NK cells exhibit heightened cytotoxic activity

Fever can be considered a form of physiological stress, as it exposes the organism to elevated temperatures in response to infection, activating thermoregulatory and immune mechanisms. This reaction primarily enhances both innate and adaptive immune responses, providing an effective defense mechanism against infections [19]. In response to infection, the pyrogenic cytokine IL-6 plays a pivotal role in the development of fever by initiating a cascade that stimulates the production of prostaglandin E2 (PGE2) in brain endothelial and perivascular cells. PGE2 then binds to the prostaglandin E receptor 3 (EP3) in the hypothalamic preoptic area, triggering downstream signaling pathways that elevate the thermoregulatory set point. In addition to IL-6, other endogenous pyrogens, such as IL-1β and TNF, contribute to fever generation by inducing cyclooxygenase-2 (COX-2) and microsomal prostaglandin E synthase-1 (mPGES-1), thereby enhancing PGE2 synthesis. This integrated cytokine–prostaglandin pathway leads to the activation of hypothalamic neurons and the subsequent initiation of heat-conservation and heat-production mechanisms. An increase of just 1 °C above 36.6 °C activates innate immune response, characterized by the mobilization of either B cells to produce antibodies and helper T cells (Th cells) to cooperate with other immune cells, and NK cells to directly destroy pathogens (Fig. 1). Furthermore, the production of pro-inflammatory cytokines promotes the recruitment of immune cells to lymphoid tissues, ensuring their precise localization and activation. In the adaptive immune response, fever enhances antigen presentation by dendritic cells (DCs) and macrophages, facilitating the activation of naïve T cells, which differentiate into effector cells capable of producing cytokines and cytolysins important in fighting infection. Fever also accelerates the proliferation of B cells, supporting the production of antibodies and the formation of immunological memory by enhancing the production of memory B cells, which mediate the secondary immune response. This coordinated response strengthens the immune system’s ability to combat pathogens effectively [19].

### Consequences of chronic stress on the immune system

As summarized in Table 1, acute stress transiently enhances immune function, including T cell proliferation and NK cell cytotoxicity, whereas chronic stress leads to dysregulation of immune responses. Specifically, prolonged exposure to catecholamines and glucocorticoids decreases T cell activation and proliferation, impairs NK cell cytotoxicity, and skews macrophage polarization toward immunosuppressive phenotypes.

**Table 1:** Comparison of acute versus chronic stress in the context of immune regulation

Aspect	Acute stress	Chronic stress	References
Duration	Short-term (minutes–hours);	Long-term (weeks–years);	[1, 2]
Physiological effects	Increased heart rate, blood pressure; enhanced alertness; glucose mobilization; catecholamine surge (epinephrine, norepinephrine); transient metabolic shift	Hypertension; sleep disruption; insulin resistance; metabolic dysregulation; sustained cortisol exposure; impaired insulin signaling; vascular remodeling	[1, 2]
Neuroendocrine axis effects	Transient SNS and HPA axis activation; short-term increased in cortisol and catecholamines (rapid resource and immune cells mobilization) returning to baseline; negative feedback intact; elevated cortisol has adaptive effects	Dysregulated SNS and HPA axis; blunted or prolonged cortisol response; altered GR sensitivity; chronic low-grade inflammation; impaired negative feedback; disrupted circadian cortisol	[1–4]
CD4^+^ T cells	Transient increase in circulation and activity; promotes Th1 cytokine burst; temporary increase IL-2 production	Decreased activation and proliferation due to glucocorticoid-mediated inhibition of NF-κB and AP-1 signaling; impaired Th1 function (shift towards Th2); increased level of Th17; leads to lymphopenia, impaired IL-2 signaling, Th1/Th2 imbalance; suppresses IFN-γ	[44–46]
CD8^+^ T cells	Enhanced trafficking and cytotoxicity (↑perforin, granzyme B); redistribution to tissues	Reduced numbers and cytotoxicity via β2-adrenergic receptor signaling and glucocorticoid-induced apoptosis; chronic antigen (stressor) exposure (functional exhaustion); PD-1 upregulation; GR receptor resistance; impaired antiviral and anti-tumor defense	[44, 47]
Treg	Short-term stress does not strongly impact Treg balance	Conflicting data: increased frequency and suppressive function, leading to immunosuppression vs. impaired Treg function despite increased numbers, resulting in loss of tolerance and pro-inflammatory skewing, chronic stress–induced glucocorticoids and sympathetic signaling may expand Treg pools, but functional impairment can occur depending on context (e.g., microbiota, tissue microenvironment)	[48–50]
NK cells	Increased cytotoxicity and rapid mobilization to blood and tissues; adrenergic mobilization, epinephrine-driven;	Decreased numbers and reduced cytotoxicity due to β-adrenergic receptor–mediated downregulation of perforin/granzyme pathways; cortisol-mediated suppression; impairs early tumor and viral clearance	[51, 52]
B cells	No major acute changes; possible short-term antibody rise due to relocation of already synthesized antibodies; sympathetic signaling can transiently boost humoral response	Reduced proliferation and antibody secretion due to impaired T cell help and direct glucocorticoid effects; suppresses B cell lymphopoiesis and class switching; weakens adaptive humoral immunity	[17, 53, 54]
Macrophages	Conflicting data: suppressed cytokine production by splenic macrophages to protect against organ injury, vs. short-term increase in circulating pro-inflammatory cytokines (IL-1β, TNF), enhanced phagocytic activity; promotes M1 activation; temporarily enhanced innate immunity	Shift toward M2-like phenotype (IL-10↑, IL-12↓) via catecholamine and glucocorticoid signaling; impaired phagocytosis; promotes chronic inflammation and tumor-supportive microenvironment; GR desensitization	[55, 56]
Dendritic cells	Enhanced antigen presentation and migration; enhanced adaptive, antiviral immunity	Impaired antigen presentation due to decreased MHC class II and costimulatory molecule expression; tolerogenic phenotype; leads to reduced T cell priming; cortisol alters maturation and cytokine profile; corticosterone impairs MHC class I antigen presentation	[57, 58]
Cytokine profile	Transient increased IL-1β, IL-2, IL-6; IFN-γ, and TNF; primes innate cytokine release	chronic elevation of IL-6 and TNF, with suppression of IFN-γ and IL-12; NF-κB activation; GR resistance; sustains low-grade inflammation; promotes chronic diseases, tumor progression and metabolic dysfunction	[46, 59, 60]
Clinical effects	Improved alertness, focus, memory consolidation; enhances hippocampal and amygdala activity; adaptive, survival-oriented mechanisms; fight-or-flight response promotes survival under threat	Impaired memory, executive dysfunction, mood disorders; hippocampal atrophy; reduced neurogenesis; altered monoamine signaling; higher risk of cardiovascular disease, diabetes, depression, infection and cancer progression; chronic inflammation and epigenetic changes contribute to disease pathogenesis	[1–4]

Chronic stress causes an elevated increase in cortisol levels through the HPA axis. Long-term exposure to a high level of glucocorticoids is linked with a significant reduction in the number of circulating lymphocytes, inducing a loss of immune cell responsiveness, leading to stress-induced immune dysfunction. In addition to the HPA axis, chronic sympathetic nervous system (SNS) activation can lead to impaired T cell function. It has been shown that prolonged exposure to catecholamines (dopamine, adrenaline, and noradrenaline) can downregulate the expression of CD4^+^ and CD8^+^ T cell receptors (TCR), leading to decreased T cell activation and proliferation [12]. The study by Cohen et al. [20] demonstrated that stressful events may increase the risk of developing infectious illness. In a series of viral infection studies, healthy adults were exposed to a virus that causes the common cold and then quarantined to determine which of them developed an infection with illness signs. Stressful life events have been shown to increase an individual’s risk of disease.

Recent research reveals the role of the gut microbiome as a key regulator of stress and inflammation [21]. Short-chain fatty acids (SCFA) produced by the gut microbiota have been shown to reduce inflammation by promoting the generation of regulatory T cells (Tregs) expressing transcription factor Foxp3, which play a key role in limiting inflammatory responses. SCFA can act as immunoregulators as they may suppress the formation of Th17 cells in the gut, thereby reducing secretion of pro-inflammatory cytokines, driving tissue inflammation [22]. Gut microbiome, including *Bacteroides fragilis*, specifically directs maturation of immune cells by producing polysaccharides (PSA), and corrects Th1/Th2 balance to maintain proper immune response [23]. In this context, the use of probiotics may offer benefits as they promote microbiome stability and diversity, which supports immune function. It was observed that ingesting the probiotic strain, *Lactobacillus plantarum* P-8, reduces stress-related symptoms and decreases plasma levels of cortisol and pro-inflammatory cytokines [24]. Taking the above into account, all disturbances in the amount and composition of the gut microbiota lead to numerous abnormalities in the immune system.

### Chronic stress and ROS: shaping a tumor-promoting microenvironment

Chronic stress represents a persistent threat to the organism and is closely associated with elevated levels of stress hormones, particularly cortisol. Sustained activation of the HPA axis and consequent glucocorticoid release promote excessive production of reactive oxygen species (ROS), leading to oxidative stress. Cortisol, acting through glucocorticoid receptors (GRs), suppresses the expression and activity of key antioxidant enzymes, such as superoxide dismutase (SOD) and glutathione peroxidase (GPX), thereby reducing the cell’s capacity to neutralize reactive oxygen species and increasing the risk of oxidative DNA and RNA damage. Excessive ROS accumulation can induce single- and double-strand DNA breaks. This damage activates apoptosis-related signaling pathways, ultimately leading to programmed cell death [25].

**Fig. 2:**
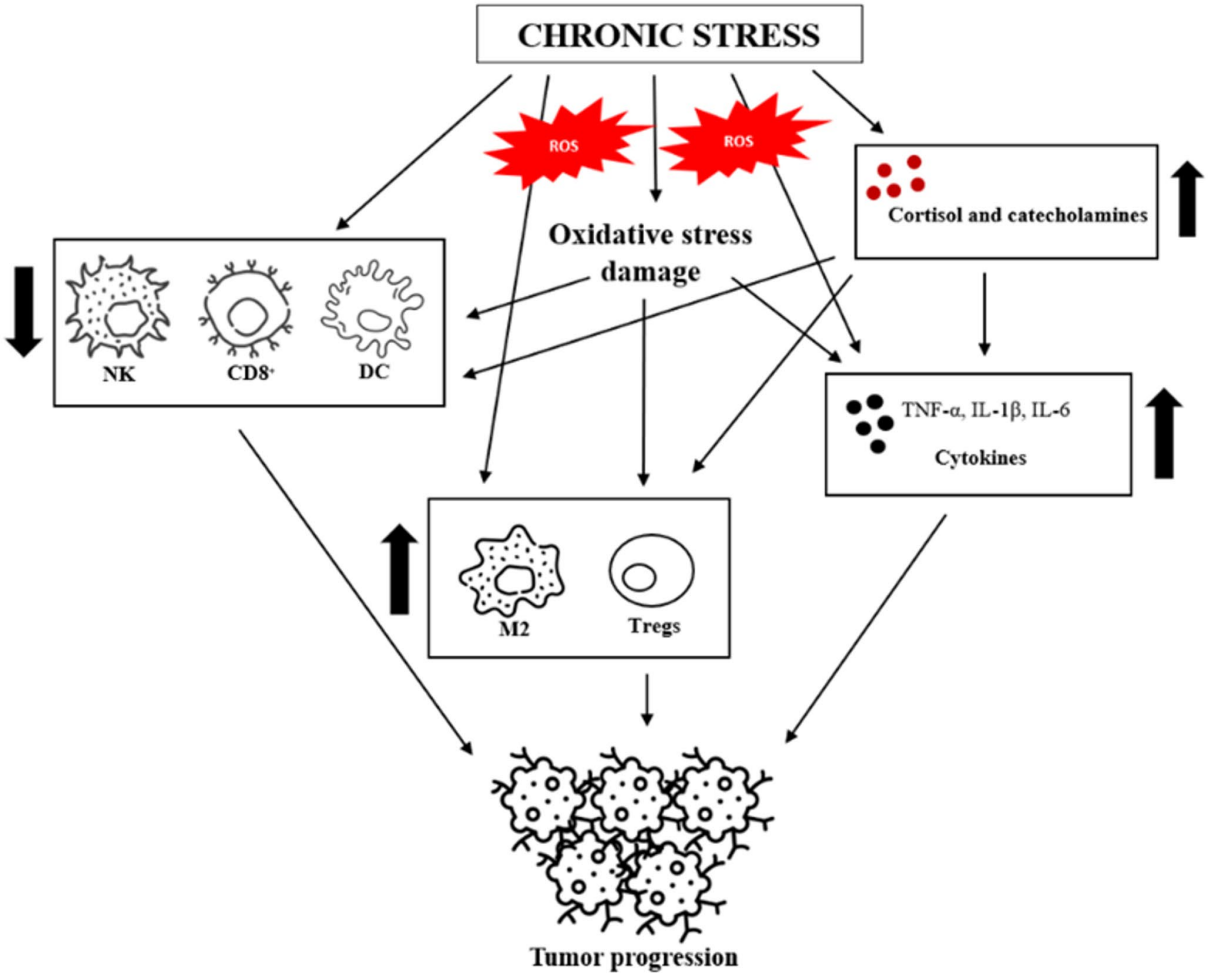
The role of chronic stress in tumor progression. The figure shows how chronic stress and oxidative stress contribute to the tumor progression. Long-term stress can increase the production of ROS and decrease the ability to neutralize them, leading to oxidative stress. Oxidative stress can exacerbate the negative effects of chronic stress, such as inflammation and cell damage. Furthermore, chronic stress induces persistently elevated levels of cortisol and catecholamines, leading to immune dysfunction characterized by inhibition of DC maturation and antigen presentation ability, as well as reduced levels and cytotoxicity of NK cells and CD8^+^ T cells. Moreover, TNF, IL-1β, and IL-6 are released, which prolongs the inflammatory state and contributes to the tumor progression. Additionally, immune cells are polarized into immunosuppressive phenotypes, such as M2 macrophages and Tregs. These processes in the immune system affect the formation of the tumor microenvironment, supporting the process of carcinogenesis

In the context of immune function, supraphysiological levels of ROS influence the production of pro-inflammatory cytokines primarily through the activation of redox-sensitive transcription factors, including nuclear factor kappa-light-chain (NF-κB) and activating protein-1 (AP-1), thereby linking oxidative stress to the modulation of immune responses. Sustained ROS production is an important factor in intracellular signaling cascades associated with the inflammatory response. It promotes NF-κB activation, which plays a role in the development of inflammatory responses by inducing the expression of multiple pro-inflammatory genes, including those encoding TNF, IL-1β, and IL-6. Excessive ROS levels were correlated with the impairment of the functionality of immune cells, inhibiting the antigen presentation ability of DCs and other antigen-presenting cells. This dysfunction is frequently accompanied by a shift toward immunosuppressive phenotypes, such as M2 tumor-associated macrophages (TAMs) and Tregs. The communication within the tumor microenvironment (TME) can be bidirectional because of TAMs, as their activity is involved in tumor progression. They secrete signal molecules such as transforming growth factor β1 (TGF-β1) and vascular endothelial growth factor (VEGF), which further promote tumorigenesis. A study on hepatocellular carcinoma (HCC) indicated that TAMs can promote stem cell-like properties by TGF-β1-induced epithelial to mesenchymal transition (EMT) in hepatoma cells [26]. Additionally, ROS-induced oxidative modifications of co-stimulatory molecules such as CD28 in CTLs and tumor-infiltrating lymphocytes (TILs) compromise their effector functions by disrupting TCR signaling and cytokine production [27]. In parallel, through the activation of transcription factors, including NF-κB, pro-tumorigenic mediators are upregulated, fostering a tumor-promoting microenvironment (Fig. 2).

Chronic stress also contributes to cancer development through neuroendocrine pathways. The increased levels of stress-related catecholamine neurotransmitters, particularly NE and epinephrine (EPI), activate β-adrenergic receptor signaling pathways, with the β2-AR subtype playing a pivotal role in tumorigenesis [28]. Stimulation of cancer cells’ proliferation by catecholamines is mediated by α- and β-ARs located on target cells. Studies in human prostate cancer (PCa) cell lines showed that NE activates β2-AR on dormant disseminated tumor cells (DTCs) within the bone marrow niche, leading to downregulation of cell cycle inhibitors: p21 and p27. Since p21 and p27 inhibit cyclin-dependent kinase (CDK) activity, their reduction releases the inhibition of cyclin-CDK complexes, facilitating tumor cell proliferation [29]. Similarly, in a gastric cancer cell line model, NE activation of β2-AR induces autophagy, which can significantly promote the proliferation of gastric cancer cells through providing metabolic substrates that allow tumor cells to survive nutrient deprivation [30]. Preclinical studies on stress revealed that increased plasma catecholamine levels and glucocorticoids enhance β2-AR-mediated upregulation of matrix metalloproteinases (MMPs) and VEGF, thereby promoting tumor progression and metastasis in a mouse model [31]. Hyperactivation of the HPA axis during chronic stress leads to increased secretion of cortisol and catecholamines, which modulate the TME by suppressing anti-tumor immune responses. Chronic stress-induced EPI elevation promotes macrophage polarization toward the M2 immunosuppressive phenotype, enhancing proliferation of tumor cells [32]. What is more, increased catecholamine levels were correlated with a decrease in NK cell-mediated cytotoxicity, impaired DCs maturation, antigen presentation ability, and T-cell exhaustion, with limitation of their ability to eliminate cancer cells [33].

Oxidative stress also contributes to tumor development through chronic inflammation. The study demonstrated that increased levels of ROS were upregulated in serum and gastric tissue samples of patients suffering from gastric cancer. ROS-mediated activation of NF-κB leads to the production of pro-inflammatory cytokines such as TNF, IL-1β, and IL-6, which further drive tumor-promoting inflammation. Persistent inflammation supports tumor growth, metastasis, and therapy resistance, with IL-6 in particular implicated in drug resistance within the TME [34]. Additionally, a major source of ROS is generated by the NADPH oxidase (NOX) family, which can act as important modulators in cancer progression. A study on non-small cell lung cancer (NSCLC) cells revealed that abundantly expressed NOX4 in NSCLC cells contributed to the cancer progression through activation of the ROS/PI3K pathway. Moreover, NOX4-mediated ROS production induced M2 polarization in macrophages and efficiently enhanced their recruitment to the tumor tissue [35].

### Challenges and future prospects

While much progress has been made in understanding how chronic stress alters immune function, translating these findings into clinical practice remains a challenge. On the therapeutic front, mindfulness-based interventions are gaining traction as effective non-pharmaceutical options for managing stress. A randomized controlled trial by Bower et al. [36] showed that mindfulness significantly reduced NF-κB activity, while enhancing transcription factors involved in type I interferon signaling. Moreover, exploratory analyses showed that patients who practice mindfulness more frequently have lower IL-6 levels at post-intervention compared to baseline levels. These findings suggest a reduction in systemic inflammation, yet the long-term durability of such effects remains unclear.

Another non-pharmaceutical approach is yoga. In fatigued breast cancer survivors with early-stage disease, yoga reduced pro-inflammatory NF-κB-related gene expression while simultaneously increasing GR activity. As individuals with more advanced disease often present with higher systemic inflammation and a distinct etiology of fatigue, this selective cohort limits generalizability. Broader trials including patients with advanced cancers are needed to clarify whether yoga-based interventions can meaningfully impact systemic inflammation and immune responses across diverse clinical settings [37].

Beyond behavioral approaches, pharmacological strategies have also been explored. GR antagonists such as mifepristone may restore normal HPA axis activity by blocking excessive glucocorticoid activity. In patients with psychotic major depression (PMD), mifepristone treatment was associated with improvements in well-being [38]. Nevertheless, this study included only a small group of patients and provided limited insight into the broader benefits of GR antagonists, leaving unanswered question about cortisol dynamics effects and HPA axis function after mifepristone therapy.

Selective serotonin reuptake inhibitors (SSRIs), including fluoxetine and sertraline, have shown preclinical potential to reverse stress-induced immune suppression. Studies indicate that these agents can regulate mRNA expression of cell cycle-related proteins, reduce stress-induced MMPs, increase the level of MMP inhibitors and tissue inhibitors of metalloproteinases (TIMPs), and enhance the cytotoxic activity of NK [39]. Such pleiotropic effects highlight their possible role in counteracting stress-driven tumor progression. Nevertheless, there is a lack of robust clinical studies, and questions regarding dosage, duration of treatment, and interactions with standard cancer therapies remain unanswered.

β-adrenergic signaling has also emerged as an important therapeutic target. Propranolol, a non-selective β-AR antagonist, has demonstrated its ability to inhibit tumor cell proliferation by lowering *ADRB2* expression and triggering caspase-dependent apoptosis pathways in liver cancer cells [40]. A study in breast cancer patients demonstrated that combining propranolol with chemotherapy enhances treatment efficacy by potentiating the anti-proliferative effects of chemotherapeutic drugs and augmenting angiogenesis inhibition [41]. Furthermore, preclinical studies revealed that propranolol can reduce the recruitment of myeloid-derived suppressor cells (MDSCs) into the TME, thereby limiting immunosuppression. These findings suggest that β-blockers may provide dual benefits in reducing both tumor growth and stress-induced immune dysfunction, although definitive large-scale trials are still needed [42].

Antioxidants represent another potential avenue. γ-tocopherol, a major isoform of vitamin E, may reduce oxidative stress by neutralizing ROS and terminating lipid peroxidation. It has also been shown to induce apoptosis in tumor cells by activating JNK/CHOP/DR5 pro-apoptotic signaling pathway, triggering caspase-8/−9-dependent apoptosis [43]. Moreover, γ-tocopherol increased levels of cellular ceramides, lipids known to be important signaling molecules that regulate apoptosis. By modulating both oxidative stress and apoptotic signaling, antioxidants could complement existing cancer therapies, but their precise mechanisms and clinical relevance remain poorly defined.

Considering the starkly different immune responses triggered by acute versus chronic stress, future research should focus on unraveling these pathways. Importantly, existing evidence is often contradictory, with some studies reporting reductions in pro-inflammatory cytokines such as IL-6 and TNF following mindfulness- or yoga-based interventions, while others observed no significant effects, underscoring the need for larger, well-controlled trials to clarify these discrepancies. Creating therapies that can restore immune balance after stress, such as β-AR antagonists, SSRIs, and targeted cytokine modulation, could be a valuable addition to standard cancer treatments. However, turning these scientific insights into lasting clinical benefits will necessitate well-structured, long-term, randomized trials that assess both overall immune responses and tumor-specific immunity.

## Conclusion

Acute stress as well as chronic stress have an impact on the immune system. The short bursts of stress can actually boost immune surveillance by quickly mobilizing innate immune cells, like NK cells and neutrophils, and redistributing effector memory T cells through catecholamine-driven β-adrenergic signaling. This temporary boost in immunity seems to prepare the body for immediate threats by ramping up cytotoxic activity and increasing adhesion molecules on lymphocytes. In turn, chronic stress profoundly disrupts immune homeostasis through prolonged activation of the HPA axis and the SNS, which leads to ongoing release of glucocorticoids and catecholamines. This can suppress the maturation of antigen-presenting cells, a shift towards the anti-inflammatory M2 macrophage phenotype, expand Treg cells, and hinder the function of cytotoxic CD8^+^ T cells. Mechanistically, long-term adrenergic stimulation through β2-AR can fuel tumor growth by increasing levels of VEGF, IL-6, and members of the MMP family, which changes how the extracellular matrix is remodeled and aids in metastasis. Moreover, the NF-κB and STAT3 signaling pathways remain constantly activated in the TME, perpetuating pro-tumor inflammation. In summary, the opposing effects of stress illustrate the dynamic and context-dependent nature of its influence on the immune system.

## Data Availability

No datasets were generated or analysed during the current study.

## Abbreviations

ACTH: Adrenocorticotropic hormone
AP-1: Activator protein-1
ARs: Adrenergic receptors
β2-AR: β2-adrenergic receptor
CDK: Cyclin-dependent kinase
CORT: Corticosterone
COX-2: Cyclooxygenase-2
CRF: Corticotropin-releasing factor
CRP: C-reactive protein
CTLs: Cytotoxic T lymphocytes
CVD: Cardiovascular disease
DCs: Dendritic cells
DTCs: Dormant disseminated tumor cells
EMT: Epithelial to mesenchymal transition
EP3: Prostaglandin E receptor 3
EPI: Epinephrine
GM-CSF: Granulocyte-macrophage colony-stimulating factor
GPX: Glutathione peroxidase
GRs: Glucocorticoid receptors
HCC: Hepatocellular carcinoma
HPA: Hypothalamic pituitary–adrenal axis
HRV: Heart rate variability
IL-1β: Interleukin-1 beta
IL-6: Interleukin 6
INF-γ: Interferon gamma
MAPK: Mitogen-activated protein kinase
MDSCs: Myeloid-derived suppressor cells
MKP-1: Mitogen-activated protein kinase phosphatase 1
MMPs: Matrix metalloproteinases
mPGES-1: Microsomal prostaglandin E synthase-1
NE: Norepinephrine
NF-κB: Nuclear factor kappa-light-chain
NK: Natural killer cells
NOX: NADPH oxidase
NSCLC: Non-small cell lung cancer
PCa: Prostate cancer
PGE2: Prostaglandin E2
PMD: Psychotic major depression
PSA: Polysaccharides
ROS: Reactive oxygen species
SAM: Sympathetic-adrenal-medullary
SBP: Systolic blood pressure
SCFA: Short chain fatty acids
SNS: Sympathetic nervous system
SOD: Superoxide dismutase
SSRIs: Selective serotonin reuptake inhibitors
TAMs: Tumor-associated macrophages
TCR: T cell receptor
TGF-β1: Transforming growth factor β1
Th cells: Helper T cell
TILs: Tumor-infiltrating lymphocytes
TIMPs: Tissue inhibitors of metalloproteinases
TME: Tumor microenvironment
TNF: Tumor necrosis factor
Tregs: Regulatory T cells
VEGF: Vascular endothelial growth factor
